# Heart rate turbulence measurements in patients with dipper and non-dipper hypertension: the effects of autonomic functions

**DOI:** 10.3906/sag-2105-177

**Published:** 2021-09-29

**Authors:** Çağlar ALP, Mehmet Tolga DOGRU, Vahit DEMİR

**Affiliations:** 1 Department of Cardiology, Faculty of Medicine, Kırıkkale University, Kırıkkale, Turkey; 2 Department of Cardiology, Yozgat City Hospital, Yozgat Turkey

**Keywords:** Electrocardiography, ambulatory blood pressure, heart rate, hypertension, autonomic nervous system

## Abstract

**Background/aim:**

Hypertensive patients have shown autonomic dysfunction that is closely associated with the measurements of heart rate variability (HRV) and heart rate turbulence (HRT). We aimed to show the alterations of HRV and HRT measurements in patients with both dipper and non-dipper hypertension.

**Materials and methods:**

This was a retrospective study consisting of one hundred and twenty-three participants (mean age ± SD, 55.7 ± 14.8 years; range, 18–90 years). The participants were divided into two groups: Group1: The patients with dipper hypertension, Group2: The patients with non-dipper hypertension. Two cardiologists performed HRV and HRT using 24-h electrocardiography (ECG) Holter and ambulatory blood pressure monitoring (ABPM) of patients.

**Results:**

The results indicated that patients in group 2 had higher low frequency power/high frequency power ratio (LF/HF), lower high frequency power (HF), root mean square of standard deviation (RMSSD) values than group 1 (p = 0.007, p = 0.008, and p = 0.002, respectively). Group 2 also showed higher heart rate turbulence onset (HRTTO) and lower heart rate turbulence slope (HRTTS) values than Group 1 (p = 0.004, p = 0.001, respectively). We performed multivariate analysis and observed that HRTTS and HRTTO have statistically significant associations with the presence of dipper or non-dipper hypertension [F = 7.755, p = 0.001], LF/HF [F = 7.868, p = 0.001], and HF [F = 4.081, p = 0.020].

**Conclusion:**

This study shows a statistically significant difference in HRT measurements between dipper and non-dipper hypertensive patients. Deteriorated autonomic circadian rhythm and autonomic functions may contribute to these results.

## 1. Introduction

Hypertension (HT) is one of the most significant cardiovascular disorders with a wide spectrum of clinical findings and complications [1]. Ischemic heart diseases, arrhythmias, cardiomyopathies, strokes, and renal failure are closely associated with HT [2]. Several studies have documented that hypertensive patients had increased target organ damage, morbidities, and mortality rates, especially in the non-dipper hypertensive patients [3–5].

Several studies have highlighted a close association between the mortality rates that also involve sudden cardiac death (SCD) and autonomic dysfunction [6]. Autonomic dysfunctions are found closely associated with the measurements of heart rate variability (HRV) and heart rate turbulence (HRT) [6,7].

In daily clinical practice, the evaluation of autonomic functions is performed by using HRV measurements [8]. There are well-known HRV measures that have indirectly shown different autonomic nervous system components. High-frequency (HF) power is used for the evaluation of parasympathetic (PS) activity, whereas low-frequency power (LF)/high-frequency power (HF) ratio is for evaluating sympathetic (SP) activity and sympathovagal balance [8].

Numerous studies have revealed an increased LF/HF ratio and decreased HF power in hypertensive patients [8,9]. Increased sympathetic activity and a decreased parasympathetic activity are closely related to increased prevalence of cardiac arrhythmia and SCD risk in many cardiovascular pathological conditions [8–11]. Besides, studies on autonomic functions of hypertensive patients showed that the patients with non-dipper hypertension had an overactive sympathetic system than those of other groups [10,11].

HRT measures are one of the most important methods for the detection of SCD risk in many clinical conditions like acute coronary syndromes and heart failure [12]. HRT is introduced as the percentage of the difference between pre-and post- two R-R intervals of ventricular extrasystole. Parasympathetic and sympathetic tonus may be associated with HRT. Several studies have shown a close association of increased turbulence onset (TO) and decreased turbulence slope (TS) measures with increased risk of SCD [7,13,14].

In this study, we attempted to investigate HRT and HRV measurements in different blood pressure (BP) levels and patterns in hypertensive patients showing dipping or nondipping characteristics.

## 2. Materials and methods

The recordings of three hundred and fifty patients, admitted from June 2016 to January 2020 to the Cardiology Department of Kırıkkale University Faculty of Medicine and also to the Cardiology Department of Bozok University with newly diagnosed hypertension and related symptoms, were evaluated. The protocol and design of this retrospective study were approved by the Institutional Ethics Committee (Research Project approval number: 2020.06.03).

### 2.1. Patient selection

The patients were selected by evaluating their medical history and using all recordings about their anamnesis, physical examination findings, BP measurements, laboratory data, radiological reports, and 24-h electrocardiography (ECG) (Holter) recordings.

Exclusion criteria were acute or chronic ischemic heart diseases, systolic heart failure congenital, and/or acquired heart diseases (containing constitutional, valvular, myocardial, and electrophysiological pathologies), peripheral artery disease, Raynaud’s syndrome, connective tissue disorders, aortic aneurysms, chronic renal insufficiency, hyperlipidemia, diabetes mellitus, all types of malignancies, morbid obesity, pulmonary diseases, pregnancy or lactation, psychiatric and neurological diseases, endocrinological diseases, drug abuse, and alcohol addiction.

### 2.2. Cardiologic evaluation

Detailed anamnesis and physical examinations of the participants were done. BP measurements were done in both arms by using a sphygmomanometer. In addition, 12-channel electrocardiography (ECG) recordings [8] and transthoracic echocardiography (Ge-Vivid 7 Pro, General Electric; FL, USA) were performed [16].

A total of 123 participants (age range,18–90 years, mean age ±SD: 55.7±14.8 years; 56 male patients, mean age ± SD: 57.2 ±16.0 years; 67 female patients, mean age ± SD: 54.4 ±13.8 years) were recruited in the study.

After recruiting the study population, the first in-office BP measurements of the patients were recorded and 24-h ambulatory blood pressure monitoring of all 123 participants were analysed. And then, all participants were grouped according to circadian blood pressure pattern and decrease in nocturnal BP levels. During the night, patients with blood pressure drop > 10% were classified as “dipper hypertension” and the rest as “non-dipper hypertension” patients (2–5).

We grouped the patients as follows:

- Group 1: Patients with dipper hypertension (DPHT).
- Group 2: Patients with non-dipper hypertension (NDPHT).

After grouping the patients as dipper and non-dipper, we analysed the 24-h electrocardiography (ECG) recordings (Holter) of the patients.

### 2.3. Heart rate variability (HRV) analysis

HRV measurements were obtained from 24-h Holter (ECG) recordings (Delmar-Impresario Medical Systems, Irvine, California, USA). Data were analyzed by using the European Society of Cardiology (ESC) criteria [8]. Both the time domain and frequency domain analyses were also performed as per ESC guidelines [8].

Time-domain HRV analysis and parameters: The proportion of NN50 divided by the total number of NN (R-R) intervals (pNN50) and its derivative, Root mean square of the successive differences (RMSSD) are associated with the parasympathetic (vagal) activity from 24-h ECG recordings [8].

Frequency domain HRV analysis and parameters: It is considered that LF power is associated with baroreflex sensitivity. Besides, HF power parasympathetic (vagal) activity is associated with the parasympathetic (vagal) activity; LF/HF ratio value indirectly indicates the sympathovagal balance. An increased LF/HF ratio indicates an excessive sympathetic activity [8].

### 2.4. Heart rate turbulence (HRT) analysis

Heart rate turbulence parameters, TO, and TS were measured by computer software (HRT View, Version 0.60–0.1Software Program, Munich, Germany). Turbulence onset (TO), a calculated value, shows the early sinus acceleration after ventricular extrasystole (VES). Calculation of TO is performed using the difference between the meantime measure of the first R-R beats in sinus rhythm following a VES and the meantime measure of the last two R-R beats in Sinus rhythm preceding a VES, divided by the mean duration of the last two sinus beats preceding the VES. A turbulence slope (TS) is defined as the maximum positive slope of a regression line assessed over five subsequent RR intervals in sinus rhythm within the first 20 sinus rhythm intervals after VES and shows the late sinus deceleration after VES [7,13–15].

### 2.5. Statistical analysis

We used SPSS version 20.0 (SPSS Inc., Chicago, IL, USA) to evaluate the data statistically. For statistical distribution of data, the normally distributed data are presented as mean ±standard deviation (SD); Student’s *t-*test was used for comparing these data. Then non-normally distributed data are expressed as median (25%–75%). Mann–Whitney U test was employed for data comparison. Partial correlation analysis, univariate and multivariate analysis of covariance (MANCOVA) were employed for evaluating statistical associations among HRTTS, HRTTO, and HRV parameters and blood pressure measurements. P-value < 0.05 was considered to be of statistical significance.

## 3. Results

There were 57 patients in the dipper hypertension group and 66 patients in the non-dipper hypertension group in the patient groups included in the study.

Table 1 shows anthropometric, biochemical, ambulatory blood pressure monitoring (ABPM) measurements of all patients. No difference in anthropometric and biochemical values among the two groups was detected.

Evaluation of BP measurements revealed no difference in-office measurements of blood pressure between the patients with DPHT and the patients with NDPHT. However, there were statistically significant differences in median diastolic BP during day time (p = 0.034), median systolic and diastolic BP of nighttime measures between the study groups (p < 0.001 and p < 0.001, respectively) (Table 1).

### 3.1. Heart rate variability and heart rate turbulence measurements obtained by the evaluation of 24-h Holter (ECG) monitoring (Table 2)

Data evaluation showed statistically significant differences in median values of minimal heart rate and the numbers of ventricular extrasystole in 24 h between the study groups ( p < 0.001 and p = 0.001, respectively) using Mann–Whitney U test.

When we evaluated the 24-h HRV test results using Mann–Whitney U test, some statistically significant differences of HF, LF/HF, pNN50, and RMSSD measures between Group 1 and Group 2 (p = 0.008, p = 0.007, p = 0.001, p = 0.002, respectively) were observed. The NDHPT patients showed a higher LF/HF ratio, which is an indicator of sympathetic activity in HRV measurements. On the other hand, there were lower HF, pNN50, and RMSSD values, which are indicators of parasympathetic activity in patients with NDHPT than those of DHPT patients (Table 2).

The results also showed that HRTTO and HRTTS measures had statistically significant differences between the patients with DPHT and the patients with NDPHT (p = 0.004, p = 0.001, respectively). The NDPHT patients showed higher HRTTO and lower HRTTS values (Table 2).

The correlations among HRV parameters (HF, LF, LF/HF, pNN50, RMSSD) and HRT measures (HRTTO and HRTTS) were also examined. The partial correlation analysis was done in order to control the effects of anthropometric measures, systolic and diastolic BP in office. A statistically significant positive correlation was found between HF and HRTTS (r = 0.314; p = 0.003), pNN50 and HRTTS (r = 0.212; p = 0.047), RMSSD and HRTTS (r = 0.226; p = 0.034). LF/HF and HRTTO (r = 0.401; p < 0.001) was observed in this study.

We also used an univariate analysis model that contains important anthropometric properties (sex, age, weight, body mass index, waist circumference), blood pressure values (systolic and diastolic), HF, LF, LFHF ratio. We detected that there are statistically significant association between HRTTO and the presence of dipper or non-dipper hypertension [F = 8.161, p = 0.005], HRTTO and HF [F = 5.151, p = 0.026], HRTTO and LF/HF [ F = 15.251, p < 0.001] (Table 3A), HRTTS and the presence of dipper or non-dipper hypertension [F = 8.803, p = 0.004] (Table 3B).

We also evaluated factors that could be associated with HRTTS and HRTTO. We used the MANCOVA model with characteristics similar to univariate analysis. HRTTS and HRTTO showed a statistically significant associations with the presence of dipper or non-dipper hypertension [F = 7.755, p = 0.001, Wilks-Lambda], LF/HF [F = 7.868, p = 0.001, Wilks-Lambda], and HF [F = 4.081, p = 0.020, Wilks-Lambda].

## 4. Discussion

In the present study, we found that the patients with NDPHT had a higher LFHF ratio, lower HF, pNN50, and RMSSD values. These findings show increased sympathetic activity in patients with NDPHT compared to the patients with DPHT. Moreover, the patients, which had nondipping blood pressure characteristics had also decreased parasympathetic activity. The patients with non-dipper hypertension showed higher HRTTO and lower HRTTS values than those with dipper hypertension.

We determined a statistically significant positive correlation between HF and HRTTS. Possibly, the parasympathetic activity might be positively correlated with HRTTS. A statistically significant positive correlation between LF/HF and HRTTO was observed. This suggests a positive correlation of sympathetic activity with HRTTS. Furthermore, the patients with non-dipper hypertension showed a greater number of VES compared to the patients with dipper hypertension.

Numerous studies have shown that patients with hypertension are not a homogenous group in clinical practice. Many clinical subtypes of hypertension have been introduced to date [17]. There are some important differences in diagnosis, clinical course, end-organ damage and complications, prognosis, clinical management, and treatment among different subtypes of hypertension [2,17–19]. Recently, patients with dipping and nondipping patterns of hypertension have drawn the utmost attention of the researchers [20,21]. The most important reason for the researchers’ interest is the presence of growing evidence about the higher incidence of target organ damages like stroke, myocardial hypertrophy and infarction, arrhythmia, and heart failure in patients with non-dipper hypertension [22–25]. Over the last decade, numerous studies were done to understand the mechanisms of dipping and nondipping patterns of hypertension [26–28]. As is well known, almost all hypertensive patients have autonomic dysfunction, including increased sympathetic activity and peripheral arterial resistance, sodium and fluid retention, increased incidence of myocardial hypertrophy, myocardial infarction, stroke, heart, and renal failure, and arrhythmias compared to non-hypertensive individuals [2, 29–31]. Besides, many studies have documented a higher incidence of clinical complications in patients with non-dipper hypertension than in patients with dipper hypertension [29–31].

Some authors have argued that there are higher sympathetic tonus and lower parasympathetic tonus in patients with non-dipper hypertension [32,33]. One study showed that the presence of myocardial repolarization abnormalities can also cause arrhythmias in patients with non-dipper hypertension [10]. An increased incidence of ventricular and supraventricular extra systoles and SCD in the same patient group have also been reported [34,35].

In clinical practice, HRT measurements are one of the most important methods to reveal the increased sudden cardiac death risk [35]. There are increased HRTTO and decreased HRTTS measures in hypertensive patients [35]. The patients with non-dipper hypertension showed more significant autonomic dysfunction, increased peripheral resistance and myocardial hypertrophy, myocardial repolarization abnormalities, myocardial ischemia, and infarction [10,29–35]. Due to these factors, sudden cardiac death risk may increase in patients with non-dipper hypertension [36–38]. Some studies have highlighted abnormalities in heart rate turbulence measurements in these patients [39]. Owing to close associations between the autonomic activity and heart rate turbulence measures, heart rate turbulence abnormalities may depend on autonomic dysfunction [35–39]. Therefore, the evaluation of HRT and HRV measurements together becomes more important.

Our findings suggest a close association between the presence of nondipping pattern in the patients with HT and abnormal HRT measures. Autonomic dysfunction shown by using heart rate variability measurements has also supported the alterations of heart rate turbulence values.

With respect to the limitations, this was a small-scale, cross-sectional, and retrospective study. Large-scale studies might be useful for the statistical evaluation of the data.

## 5. Conclusion

It may be concluded that there are significant differences in heart rate turbulence measurements between the patients with dipper and non-dipper hypertension. Increased autonomic dysfunction in patients with non-dipper hypertension may contribute to these alterations.

## Figures and Tables

**Table 1:** The differences of anthropometric, biochemical and ambulatory blood pressure measurements (ABPM) between the study groups.

	The patients with dipper hypertension n:57 mean±sd	The patients with non-dipper hypertension n:66 mean±sd	p value
**Anthropometric measurements**			
**Age (years)**	57.1 ± 16.6	54.4 ± 13.2	0.327
**Sex** **	Male: 25 Female: 32	Male: 31 Female: 35	0.730
**Weight(kg)**	76.5 ± 10.8	80.8 ± 12.7	0.052
**He** **İ** **ght(cm)**	165 ± 7	166 ± 1	0.627
**Body mass Index (kg/m** **^2^** **)**	28.24 ± 4.70	29.69 ± 5.34	0.128
**Waist circumference (cm)**	99.5 ± 14.2	100.3 ± 15.2	0.815
**Biochemical measurements**			
**Serum blood glucose level (fasting) (mg/dL)**	108.4 ± 36.3	109.5 ± 44.0	0.878
**Serum urea level (mg/dL)**	37.6 ± 33.5	25.5 ± 12.3	0.263
**Total cholestrol (mg/dL)**	203.7 ± 33.9	202.3 ± 35.7	0.830
**LDL cholestrol (mg/dL)**	123.6 ± 27.1	119.4 ± 34.8	0.510
**HDL cholestrol (mg/dL)**	50.7 ± 11.6	47.8 ± 10.8	0.197
**Trigliserid (mg/dL)**	147.6 ± 77.4	167.7 ± 82.6	0.205
**Blood pressure measurements in office**			
**Mean systolic blood pressure (mmhg)**	144 ± 14	145 ± 15	0.579
**Mean diastolic blood pressure (mmhg)**	85 ± 10	86 ± 9	0.585
**24-h ambulatory blood pressure monitoring (Abpm) measurements**			
**24-h median systolic blood pressure (mmhg)** *	140 (132–149)	144 (133–152)	0.201
**24-h median diastolic blood pressure (mmhg)** *	82 (76–88)	88 (79–95)	**0.008**
**Daytime measurements (06:00–22:59)**			
**Median systolic blood pressure (mmhg)** *	148 (139–155)	146 (134–152)	0.201
**Median diastolic blood pressure (mmhg)** *	83(79–92)	90(81–97)	**0.034**
**Nighttime Measurements (23:00–05:59)**			
**Median systolic blood pressure (mmhg)** *	127(120–136)	140(132–151)	**<0.001**
**Median diastolic blood pressure (mmhg)** *	76(70–81)	84(76–91)	**<0.001**

Student T test, Mean±SD, p < 0.05,

* Mann–Whitney U test, median (%25–%75),

** Pearson chi-square, p < 0.05.

LDL: low density cholesterol.

HDL: High density cholesterol.

**Table 2:** The differences of Hrv and Hrt measurements between the study groups.

	The patients with dipper hypertension N:57 mean±sd	The patients with non-dipper hypertension N:66 mean±sd	p value
**24-h Ecg Holter monitoring and heart rate variability (Hrv) measurements**			
**24-h heart rate monitoring measurements**			
Mean heart rate* **(L/min)**	75.00(65.00–80.50)	75.50(70.75–82.25)	0.155
Min heart rate* **(L/min)**	46.00(42.00–52.50)	52.00(48.00–55.00)	**<0.001**
Max heart rate* **(L/min)**	135(115–150)	133(21–149)	0.835
**Mean r-r interval** * **(msn)**	821(740–930)	789(720–850)	0.127
**Ves 24** *	191(56–943)	667(179–2376)	**0.001**
**Sves 24** *	178(51–856)	240(77–565)	0.270
**24-h heart rate variability (HRV) measurements median (%25–%75)**			
**Frequency domain analysis**			
**LF (ms** ^2^ **)** *	333(179–607)	254(136–527)	0.153
**HF(ms** ^2^ **)** *	137(79–294)	103(44–149)	**0.008**
**LF/HF** *	2.27(1.57–3.40)	3.00(1.91–4.80)	**0.007**
**Time domain analysis**			
**PNN50 (%)** *	7.60(3.75–18.35)	4.65(1.43–8.68)	**0.001**
**RMSSD (ms)** *	29.00(23.00–42.50)	25.00(17.50–31.50)	**0.002**
**24-h heart rate turbulence (hrt) measurements median (%25–%75)**			
HRT-TO* **(%)**	−0.61[(−2.00)−(+2.00)]	0.13[(−0.56)−(+4.00)]	**0.004**
HRT-TS* **(msn/beat)**	6.48 [(4.51)–(10.23)]	3.26[(2.00)–(7.05)]	**<0.001**

Student T test, Mean±SD, p < 0.05,

* Mann–Whitney U Test, Median (%25–%75), p < 0.05.

VES 24 : The numbers of detected ventricular extrasystole in 24-h ECG holter monitoring.

SVES 24: The numbers of detected supraventricular extrasystole in 24-h monitoring.

HRT-TO: Heart rate turbulence onset.

HRT-TS: Heart rate turbulence slope.

LF:Low Frequency power.

HF: High Frequency power.

LFHF:LF/HF RATIO.

PNN50: Normal RRintervals >50 ms computed over the 24-h ambulatory ECG (Holter recordings).

RMSSD: Root mean square of the successive differences.

**Table 3a:** Univariate analyses. The associations of Hrtto and other parameters.

Parameters	F	p value
**Age**	1.073	0.303
**Sex**	0.413	0.522
**The presence of dipper or non-dipper hypertension**	8.161	**0.005**
**Height**	0.367	0.546
**Weight**	0.266	0.607
**Body mass index**	0.140	0.709
**Waist circumference**	0.027	0.871
**Low frequency power(Lf)**	0.187	0.667
**High frequency power(Hf)**	5.151	0.026
**Lf/Hf ratio**	15.251	**<0.001**
**Systolic blood pressure**	0.010	0.920
**Diastolic blood pressure**	0.277	0.600

Univariate analysis, p < 0.05.

Lf/Hf Ratio : Low frequency power/ high frequency power.

**Table 3b:** Univariate analyses. The associations of Hrtts and other parameters.

Parameters	F	p value
**Age**	3.082	0.083
**GenSexder**	0.044	0.835
**The presence of dipper or non-dipper hypertension**	8.803	**0.004**
**Height**	2.199	0.142
**Weight**	2.555	0.114
**Body mass index**	2.405	0.125
**Waist circumference**	0.172	0.680
**Low frequency power(Lf)**	0,001	0.992
**High frequency power (Hf)**	2.481	0.119
**Lf/Hf ratio**	0.256	0.614
**Systolic blood pressure**	0.042	0.839
**Diastolic blood pressure**	0.288	0.593

Univariate analysis, p < 0.05

Lf/Hf Ratio : Low frequency power/ high frequency power.
